# VExUS: common misconceptions, clinical use and future directions

**DOI:** 10.1186/s13089-024-00395-0

**Published:** 2024-11-26

**Authors:** Philippe Rola, Korbin Haycock, Rory Spiegel, William Beaubien-Souligny, Andre Denault

**Affiliations:** 1 Assistant Professor, University of Montreal, Chief of Service, Intensive Care Unit Santa Cabrini Hospital, CEMTL, Montreal, Canada; 2 Assistant Professor, Loma Linda University, School of Medicine, Loma Linda, USA; 3 Department of Emergency Medicine, Riverside University Health System and Loma Linda University, Moreno Valley/Palm Springs, California USA; 4 Departments of critical care and emergency medicine, Medstar Washington Hospital Center, Washington, SC USA; 5 Innovation hub, Centre de recherche du Centre Hospitalier de l’Université de Montréal, Montréal, Quebec Canada; 6 Division of Nephrology, Centre Hospitalier de l’Université de Montréal, Montréal, Quebec Canada; 7 Département d’anesthésiologie et de médecine de la douleur, Directeur du programme de Fellowship en échographie ciblée, Institut de Cardiologie de Montréal, Montreal, Canada

## Abstract

There has been a significant interest in venous congestion in recent years, among which the VExUS score has been prominent, both in clinical practice and research efforts. We have noted some recurrent misconceptions among clinicians which are also reflected in certain research efforts. Notably, the misguided attempt to correlate VExUS to volume status, which is only one of the factors influencing it, as well as attempts to re-interpret VExUS in the context of certain pathologies, which reflects a fundamental misunderstanding of its circulatory perspective. In this article we review the physiological basis of the VExUS assessment as a measure and marker of venous congestion from the organs’ standpoint and its role as part of the emerging concept of fluid tolerance, in hopes to address these misconceptions for clinicians and for important further studies.

## Introduction

In 2020 we derived and published a tool using bedside ultrasound to quantify the degree of venous congestion in critical ill patients. This tool, Venous Excess by UltraSound (VExUS) is the combination of inferior vena cava (IVC) ultrasound, hepatic venous, portal venous and intrarenal venous Doppler envelopes, all previously used to assess venous congestion in isolation [1]. These solid organ Doppler patterns, using a 3 point severity grading in the presence of a plethoric IVC, generated a score ranging from 0 to 3, and, in a cohort of post-cardiac surgery patients was associated with markedly increased risk of acute kidney injury (AKI) [2]. In the years since our initial publication, we have been overwhelmed by the incredible amount of discussion and research it has generated. We commend all those who have dedicated time and effort towards moving the needle forward on venous congestion and expanding the patient populations in which VExUS can point to potential organ dysfunction. Despite this evolution in our understanding of VExUS, we feel that certain misconceptions sometimes emerge and warrant clarification.

## Methods

In order to determine the important points to address, the authors identified a number of recurrent misconceptions noted in the course of clinical teaching, workshops and presentations’ question and answer sessions, as well as discussion with clinical peers.

## VExUS and volume status

VExUS is often referred to as a tool to quantify a patient’s volume status. Firstly, we would like to discourage the use of the term volume status, which is an exceedingly vague concept that is often understood differently by different physicians. For example when someone uses the term volume status, it is unclear whether they are referring to intravascular volume, both intra-and extravascular volume, stressed or unstressed volume, etc. There has been a longtime love affair with the somewhat quixotic quest for a “fuel gauge” of intravascular volume which started with the central venous pressure (CVP), then the superior and inferior vena cava (SVC/IVC), and now, in many clinicians’ minds, the VExUS score. This exposes a certain misunderstanding or hopeful attempt to shortcut the non-linear relationship that exists between volume and pressure. While they are obviously related, in a multi-compartmented system with varying elastances, it is simply impossible to use pressure as a measure for volume - or vice-versa: it is time for clinicians to let go of this dream. All the above failed as simple gauges of “volume status”, through no fault of their own, but rather because the question asked was an impossible one.

**Fig. 1:**
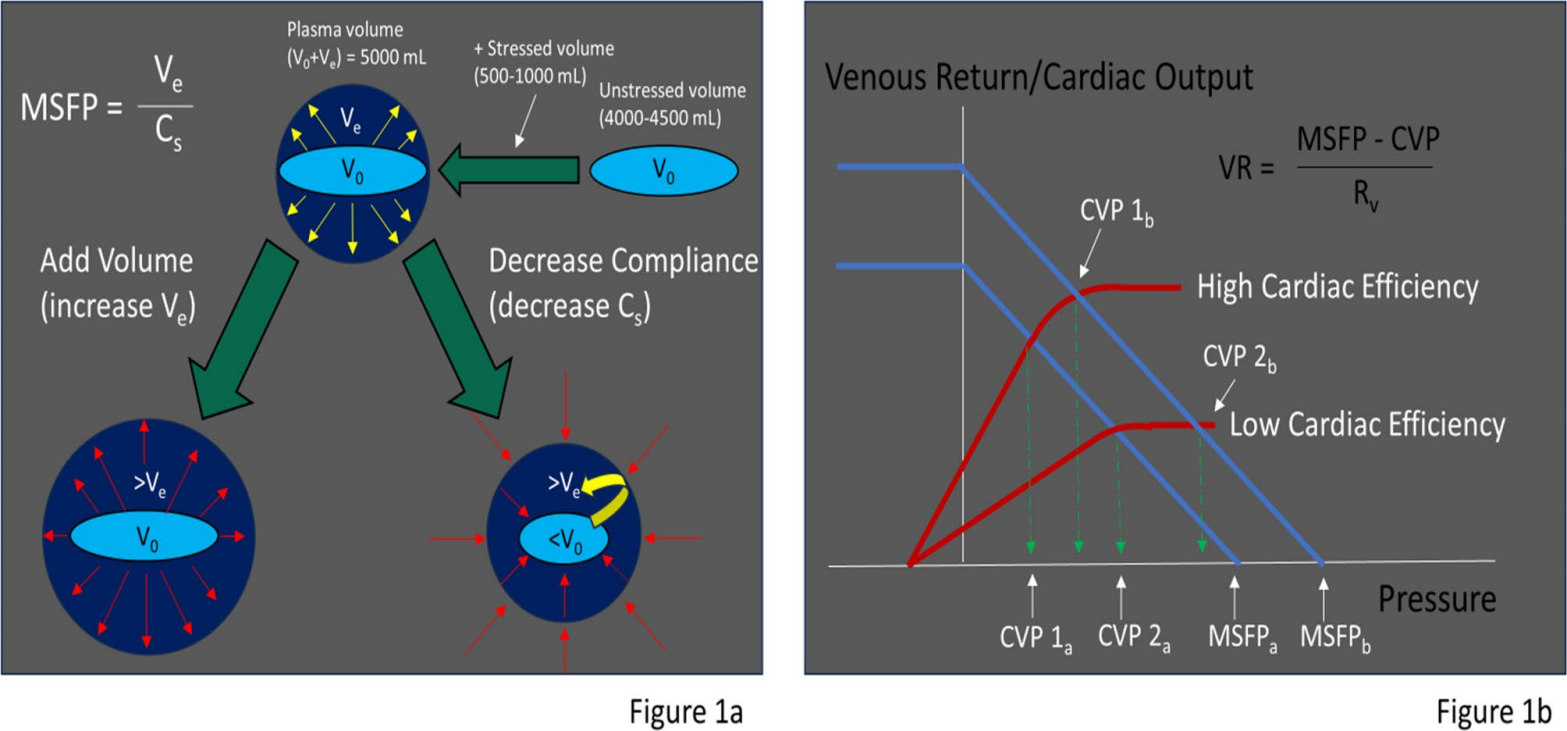
**1a**: Intravascular volume is composed of unstressed volume that does not distend the vascular walls and stressed volume that distends the walls resulting in elastic recoil of the walls generating the MSFP. MSFP increases with increased stressed volume (where the walls are further distended) and decreased compliance (where the vessels become stiffer and unstressed volume is converted to stressed volume). **1b**: The CVP depends on the cardiac efficiency and the MSFP. Venous return is driven by the difference in MSFP and the CVP. When CVP = MSFP venous return will be zero. Increases in the MSFP or decreases in the cardiac efficiency will increase CVP, but the CVP and cardiac output depends on the operating point where the venous return line and Frank-Starling curves intersect. V^0^= unstressed volume, V^e^=stressed volume, C^s^=systemic vascular compliance, V^r^=resistance to venous return, VR= Venous Return

So, what does VExUS truly measure? It reflects the ever changing relationship between the upstream venous pressure (mean systemic filling pressure (MSFP) and right atrial pressure. The MSFP, crucially, depends on two distinct factors that interact to determine this pressure. Firstly, there is the volume that “stretches” the walls of the collective blood vessels leading to elastic recoil creating pressure (as opposed to unstressed volume which is considerable, but does not distend the highly compliant venules), and secondly there is the degree of compliance in the blood vessels. The less compliant the blood vessels, the more a stressed volume will generate a pressure. The CVP depends on the magnitude of the MSFP and the efficiency of the right ventricle (RV), which when working well will keep the CVP low. The venous return (VR)--which equals the cardiac output in the steady state–depends on the difference between the MSFP and the CVP. (Figure 1a and b)

**Fig. 2:**
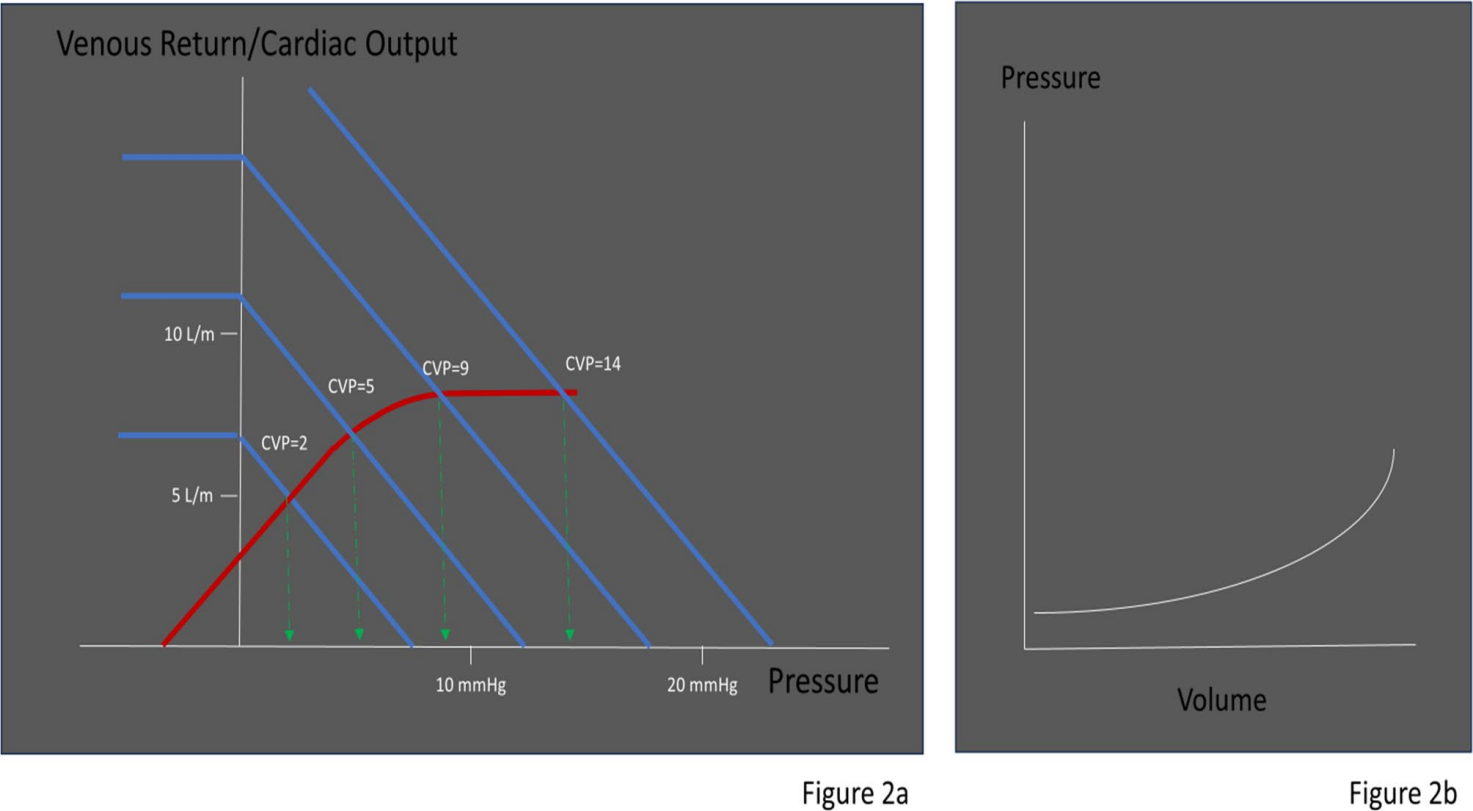
**2a:** A normovolemic person is infused with repeated boluses of crystalloid, thus raising the MSFP. Initially the response is to increase the VR with only small increases in the CVP. However, once the limit of RV compliance is reached the CVP and MSFP rapidly rise with little increase in VR. The rise in MSFP results in venous congestion of the organs and limits perfusion. **2b**: Relationship of venous compliance. Initially, additional stressed volume raises the venous pressures minimally until the limits of compliance is reached. At that point the venous pressures rapidly rise with addition of even small amounts of volume

In a patient who is initially normovolemic but infused with enough fluids, we will observe a rise in their MSFP which in turn will lead to a rise in CVP. If continued, this indiscriminate fluid administration will increase preload until the right ventricle is anatomically incapable of accommodating further volume expansion, and CVP will start to rise sharply [3]. (Fig. 2a) The venous system–having a large capacitance–is forgiving to a point. This point is the limit of venous compliance, where PMSF will then quickly rise, preserving VR at the expense of pathologically increased capillary hydrostatic pressures. (Fig. 2b) This will eventually result in a plethoric IVC and transmission of pressure waves upstream, causing abnormal Doppler envelope changes and an elevated VExUS score. In such a case, VExUS will correlate to intravascular volume.

**Fig. 3:**
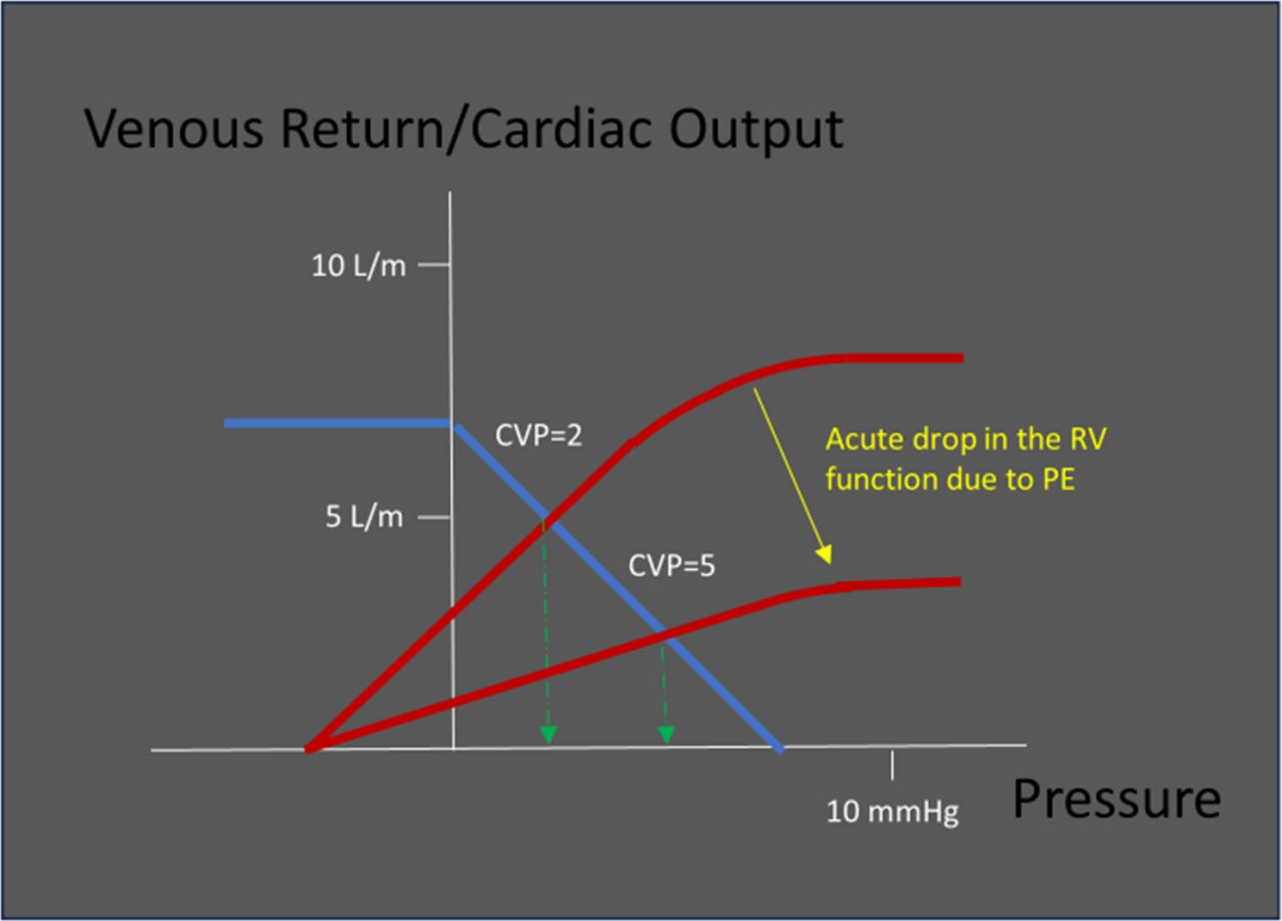
An acute rise in RV afterload due to a pulmonary embolism causes a significant decrease in RV efficiency. The VR drops whilst the CVP rises. Any attempt at a volume challenge will most likely result in an increase in the CVP and MSFP without a significant increase in the VR

Now take for example a patient who develops a large pulmonary embolism, leading to a acute increase in pulmonary arterial pressure and marked right ventricular failure, the CVP will rise due to the inability of the RV to handle its afterload, and, if sufficiently elevated, will result in increasing VExUS scores. Without an additional rise in intravascular volume. (Fig. 3)

**Fig. 4:**
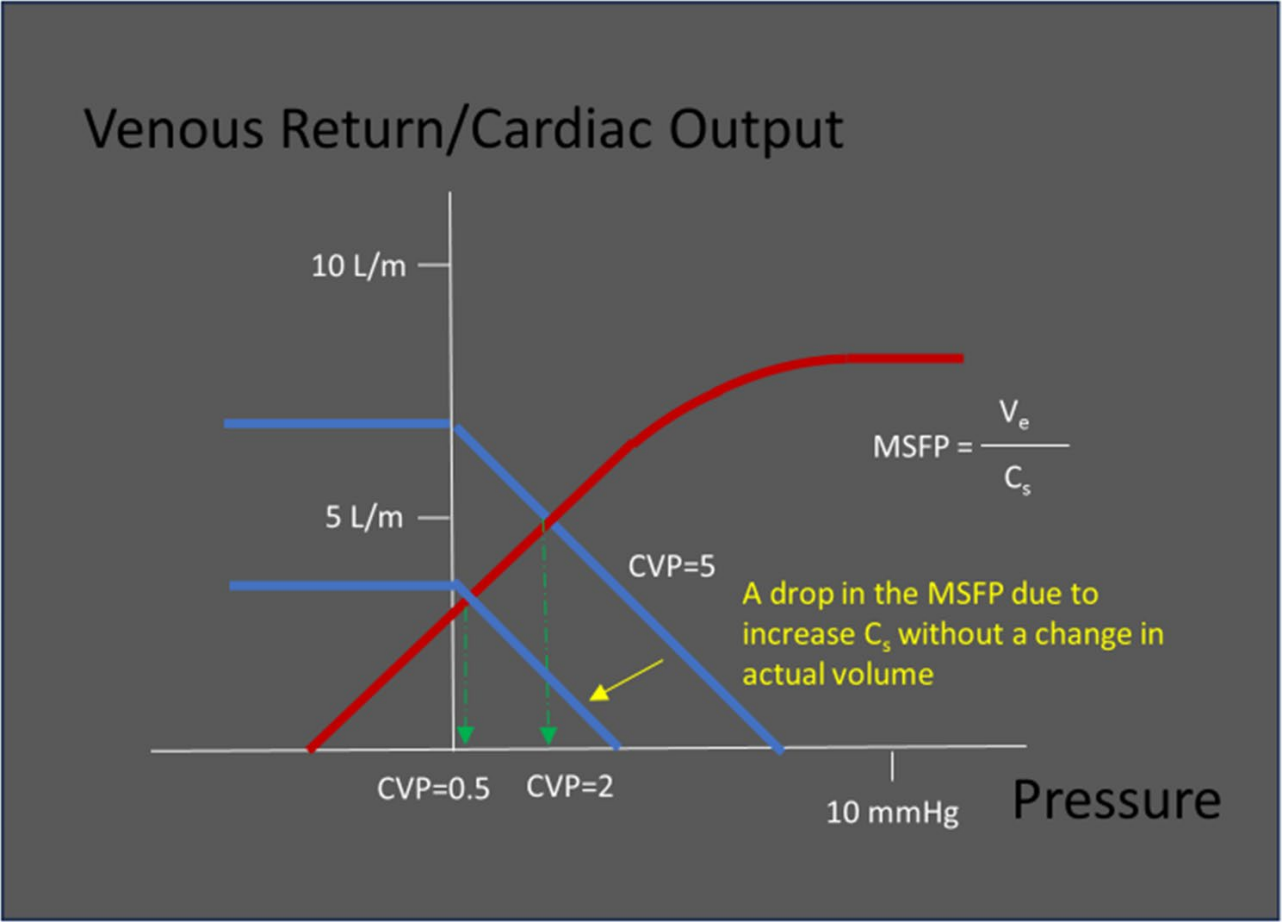
An acute rise in systemic vascular compliance (without a change in volume) results in a decrease in MSFP, CVP, and VR. Administration of either a vasopressor or a crystalloid bolus will shift the MSFP toward normal. However, note that the original drop in MSFP was independent of actual volume, and was instead a result of a change in vascular compliance alone

In a third potential scenario, a patient presents with septic shock. Here, the vasodilation will lead to a decrease in MSFP and thus venous return, with an inevitable drop in CVP. This would decrease IVC size and - if congestion had been present, VExUS score. No change in intravascular volume once again. (Fig. 4) Conversely, as this patient improves, resolving vasoplegia will increase MSFP and thus venous return and congestive indices may appear, also without a change in intravascular volume. Septic shock and RV failure can of course co-exist, with the resulting VExUS score reflecting a balance between the degree of vasodilatation and RV failure.

So, for instance, following the stable outpatient with congestive heart failure (CHF) and no other active process, a change in VExUS score is likely to reflect a change in the intravascular volume and perhaps extracellular water. In support of this, Husain-Syed et al. showed a correlation between overhydration determined by bioimpedance analysis and worsening intra-renal venous Doppler patterns in a cohort of non-critically ill right heart failure patients [4]. 

So can the VExUS score be used to assess intravascular volume at all? Yes, but only in the same patient without change in vascular compliance or RV-PA coupling (i.e. a decrease in RV efficiency). In the critically ill patient? Not really. With regard to VExUS, intravascular volume is one part of a comprehensive interlinked system.

## “Interpreting” VExUS under different clinical scenarios

As elucidated by the above examples it is apparent that the VExUS score, like its predecessors, cannot be thought of as a simple dichotomous tool to identify volume status. Rather its findings must be interpreted in the clinical context of the patient in whom the exam is being performed. Given this admonition,“how does one interpret VExUS findings in the presence of tricuspid regurgitation (TR), right ventricular dysfunction, mechanical ventilation, etc…” (or other conditions that alter CVP without necessarily increasing volume), the answer is actually quite simple and often becomes apparent as the experience of the operator grows: there should be no change at all in the *interpretation* of the VExUS score.

What does this mean? That one should not dismiss an elevated VExUS score on the basis of the presence of one of these factors, e.g. “I don’t have to worry about the VExUS 3, it’s just because the patient has severe TR/high PEEP/etc…”.

Here is where it is important to realize that the VExUS score - as should any measure of venous congestion - is a measure of *organ afterload* first and foremost. VExUS looks at the circulation from the organ’s standpoint looking downstream, not from a central cardiac perspective. The cause behind the afterload elevation is immaterial in the interpretation. Measuring the degree of venous congestion represents an alarm system of sorts for tissue perfusion and organ function. An abnormal VExUS score tells the clinician that there is a problem above but it does not tell what the problem is. An abnormal VExUS scan can be thought of as a symptom. It behooves the clinician to identify the underlying cause behind these Doppler findings. In the setting of a critically ill patient, this typically includes a more thorough bedside echocardiographic exploration in the hopes of identifying the potential causes of the abnormal VExUS findings.

It is obviously beyond the scope of this - or any single - article to address every management strategy of the myriad of pathologies that result in an elevated CVP. Therefore, the role of the VExUS score is to tell the clinician to grab the ultrasound probe, find the etiology and personalize the management. Do not treat a VExUs score, treat the patient.

## So what DOES VExUS really measure?

It is a measure of the severity of venous congestion, and it has been demonstrated that higher levels are associated with the potential development of organ dysfunction. Along with other tools, sonographic and otherwise, VExUS can help establish the degree of fluid tolerance, a concept recently put forward by Kattan et al. [5] Fluid tolerance is a concept describing a state where the addition of additional volume is unlikely to result in harm to the system or an organ system. This is quite different from fluid responsiveness which is defined by a state where the addition of volume to the vascular compartment will result in an increase in the SV or cardiac output (CO). Prior to the proposal of fluid tolerance, the primary strategy recommended to combat the harms of over-resuscitation was utilizing tools that assist in identifying patients who are only fluid responsive, without regard to fluid tolerance [6]. Although such strategies are preferable to indiscriminate administration of IV fluids, they do not represent the optimal strategy. Studies suggest the increase in CO in response to a fluid bolus is fleeting, lasting somewhere between 30 and 120 min in most patients [7]. Such transient increases in CO are of questionable clinical relevance.

While fluid responsiveness asks whether a fluid bolus will increase a patient’s CO, a surrogate outcome of questionable clinical import, fluid tolerance asks a far more holistic question. Fluid tolerance asks clinicians to calculate the potential harms of fluid administrations and the potential benefits and to decide whether a fluid bolus is more likely to help or harm the patient in question. Rather than VExUS serving as a dichotomous test, determining when to administer IV fluids with absolute certainty, it should serve as a piece of the greater puzzle. Its findings should be interpreted in the context of the patient, their comorbidities, their current physiological perturbations, the current moment in the ongoing resuscitation, and the amount of fluid they have received up to this point. It is only then the VExUS study can be appropriately interpreted.

## Questions for future research and limitations of VExUS

We believe that further research intended to demonstrate VExUS’ as a tool to quantify volume status would be generally unhelpful. Rather, it would be of tremendous benefit for researchers to perform interventional studies comparing VExUS to conventional strategies to guide fluid resuscitation, treatments for cardiorenal syndromes, acute kidney injury, initiation of de-resuscitation efforts, to name a few.

Observational, associative and interventional studies would also be very useful looking at VExUS and markers of RV-PA coupling, as a major gap in knowledge is the targeted decongestion, particularly in patients with severe pulmonary hypertension. Empirically, we have seen patients who tolerate decongestion to a VExUS score of 0, while others suffer a loss of forward flow with worsening renal function and hypotension with a drop in portal pulsatility from 100 to 60% - technically remaining at VExUS 3. It would be fascinating to see how this relates to RV-PA coupling indices.

False positives for elements of VExUS exist. Portal vein pulsatility can be observed in thin young patients with hyperdynamic circulation and abnormalities can also occur in the context of severe cirrhosis. However, the compound nature of the score provides robustness such that most non-overlapping false positives will likely not result in a falsely elevated score. However, it is worth stating that VExUS has not been studied specifically in severe cirrhotics and there should be careful clinical consideration in its interpretation until this population has been further studied. A case of a larger, plethoric-appearing IVC has been described with a measured CVP of around 1mmhg, potentially due to liver parenchymal fibrosis resulting in a “fixed” venous anatomy (Argaiz, personal communication).

VExUS does represent a somewhat advanced “POCUS” skillset as it requires a basic competence in pulse wave Doppler ability. In addition, a physiological understanding of the reasons for the causes of false positives and false negatives is necessary to interpret some VExUS cases. Because of this VExUS may require additional training for the novice–analogous to investigating cardiac output from pulsed wave Doppler of the left ventricular outflow tract. From an educational standpoint beyond the authors’ experience, a VExUS substudy of ANDROMEDA-SHOCK 2 is currently underway, and investigators (who were already competent at general POCUS) performing the exam followed a half-day online course including an examination, and their images were reviewed by experts with considerable VExUS experience with excellent correlation, suggesting that it can be taught relatively efficiently over a short time, but requires some supervision [8]. 

As is the case for any new diagnostic method, more work needs to be done in terms of delineating pitfalls and establishing learning pathways. One of the keys to minimizing the former is to avoid using VExUS in isolation, but rather as part of a comprehensive hemodynamic assessment.

## Conclusion

It is important for clinicians using VExUS as part of their assessments to understand the fundamental principle that venous congestion studies assess organ afterload. They act as a warning system telling us that the degree of congestion is potentially deleterious to tissue-perfusion and organ function. The next step is to identify the cause using bedside ultrasound and other imaging technologies. Only then should personalized optimization be undertaken, specific to the underlying cause.

## Data Availability

Not applicable.
